# Community Navigation: Iterative Development and Implementation of a Prenatal Psychosocial System of Care

**DOI:** 10.1007/s11121-026-01939-7

**Published:** 2026-06-25

**Authors:** Helen M. Milojevich, Debra L. Best, W. Benjamin Goodman, Peter D. Rehder, Kenneth A. Dodge

**Affiliations:** 1 Sanford School of Public Policy, Duke University, 201 Science Drive #255, Durham, NC 27708, USA; 2 Department of Pediatrics, Duke University, Durham, USA; 3 American Institutes for Research, Arlington, USA

**Keywords:** Community Navigation, Home visiting, Pregnancy, Families

## Abstract

We describe the iterative process of developing a universal psychosocial system of primary care across early life, called Community Navigation, and evaluate whether it can be implemented during the prenatal period with high engagement, identification of family needs, connections to community services, and family satisfaction. Community Navigation is a manualized program to improve child and family outcomes by engaging families during pregnancy, co-identifying their family-specific needs, and connecting them with community resources to address their needs. Continuous quality improvement efforts enhanced and refined the program leading to three iterative versions (Navigation 1.0, 2.0, and 2.1). Here, we report implementation metrics across all three versions. In each version, families were recruited during pregnancy and offered 1–3 visits with a trained Navigator. Findings from Versions 1.0 and 2.0 were used to improve the Navigation protocol, resulting in Navigation 2.1. Of the 194 Navigation 2.1 families who were eligible and offered the program, 81% completed the initial Navigation visit and 82% of those families completed at least one follow-up visit prior to birth (net total completion = 65% of the eligible population). Adherence to the protocol was 93%. Nearly all families (98%) reported at least one unmet need, with 75% reporting a major need requiring a community resource connection. Connection rates were high: 66% of families who received referrals were successfully connected to at least one community agency. Families reported high satisfaction with the program. Community Navigation holds promise as a novel system of universal primary psychosocial care for birthing families.

Families of young children in the United States are struggling. Parents face numerous challenges, including rising childcare costs, increases in mental health disorders, and growing social isolation and loneliness (Herbst, 2023; Khadka et al., 2024; Nowland et al., 2021). These challenges impact parents’ ability to provide safe, stable, and nurturing environments needed for optimal child development (Frosch et al., 2021). One in five mothers experience a mental health problem during pregnancy or the postnatal period (Fawcett et al., 2019), which, in turn, increases the risk of child maltreatment (Ayers et al., 2019). Almost 3 million children are investigated annually for experiencing abuse or neglect (US DHHS, 2026), and over one third of all children born in America will have been investigated by Child Protective Services for the experience of child maltreatment before age 18 (Kim et al., 2017). Child outcomes in the USA are poor compared to other industrialized nations and were exacerbated by the COVID-19 pandemic. Despite spending more than three times more per capita on healthcare than other developed nations, the USA ranks 33 out of the 38 nations in the Organisation of Economic Co-operation and Development (OECD) in the birth-to-five mortality rate (Gumas et al., 2025). Nearly 15% of 9–24-month-olds have a developmental concern that qualifies them for early intervention service, yet only 40% of those who qualify receive services (Boyle et al., 2011). Fewer than half of 5-year-olds are ready for kindergarten (Ghandour et al., 2021). Moreover, disparities in these child outcomes exist across race and income groups, which are worsening rather than improving (Reardon & Portilla, 2016; US DHHS, 2026).

To improve the health and well-being of young children and their families, the federal government through the Maternal, Infant, and Early Childhood Home Visiting (MIECHV) Program awards roughly $500 million annually to US states and territories to provide evidence-based home visiting services to families of young children (HRSA, 2025). Although the exact curriculum differs across home visiting models, all target improving the home and family environment for children during their first years of life (Edwards & Lutz-ker, 2008; Gershater-Molko et al., 2003; Olds, 2006; Wagner & Clayton, 1999). Most home visiting programs take a long-term, intensive approach by serving volunteer families selected based on demographic risk, such as first-time, low-income mothers. One limitation of this approach is that risk is defined narrowly and, therefore, some families in need of such services may not be eligible while other high-functioning families may receive services unnecessarily. Moreover, despite federal funding to all 50 states, the District of Columbia, and five US territories, the MIECHV Program annually serves only roughly 75,000 of the 20 million families with children aged 0 to 5 (HRSA,2025). As such, the sum of these programs has not yet achieved population impact on child and family outcomes. Programs are needed that reach universally and aspire to have population impact.

A promising approach to population impact is universal home visiting, such as Family Connects. Family Connects (FC) was created to reduce population rates of child maltreatment and to promote child and family health and well-being in the early postpartum period, a time when almost all families report some need for support (Dodge & Goodman, 2019). Grounded in a developmental science understanding of how risk for adverse child outcomes accrues and a public health understanding of integrated systems of care, FC combines a “top-down” approach of engaging and aligning services supporting families of infants with a “bottom-up” approach of engaging every birthing family through nurse home visiting to assess family-specific needs. Families with needs are connected to tailored community services for ongoing support. Randomized trials indicate FC can be implemented with high penetration and fidelity and brings positive impact on improving mothers’ mental health while also reducing emergency medical care costs and child abuse investigations (Dodge et al., 2014, 2019; Goodman et al., 2021). A population-level implementation in four rural counties yielded a 32% reduction in the total number of child emergency department visits by age 24 months (Goodman et al., 2022).

To date, FC has been implemented as a population-level intervention at one point in the life course only (at birth). Feedback from participating parents and implementing communities indicates their strong endorsement of FC but also a desire to begin receiving support before birth and for ongoing connection to an interventionist throughout early childhood. Specifically, given that family needs change over time, they recommend ongoing connection through brief annual check-ins to ensure that all families get connected to the right support at the right time. They also advocate for a focus on child cognitive, social-emotional, and behavioral development from birth through kindergarten matriculation, and support to address developmentally timed challenges such as discipline, developmental screening, and parental work-family coordination. Moreover, despite evidence of long-term positive impact on maternal mental health, child maltreatment, and child emergency medical care, FC impact has not been found on sustained community connections or child social-emotional or behavioral outcomes.

To address parent and community feedback, as well as to further improve and equalize population outcomes in child development over time, a team of community leaders and prevention scientists has developed a new program called Community Navigation (CN) through an iterative process as a universal psychosocial system of primary care across early life. Building from FC, CN consists of trained Navigators who reach families during pregnancy, coordinate with the FC nurse at birth, and complete well-family visits annually until kindergarten matriculation. Navigators provide support, assess family-specific needs for both parents and the child, deliver brief interventions, and connect families with community resources and services for ongoing child development needs. Thus, CN serves as a light-touch, universal screening and triage program that connects families to resources and long-term forms of support (e.g., intensive home visiting, case management) as needed. CN also grows the network of community agencies that families can access, reflecting new and emerging family needs for support across childhood.

The process of designing CN followed pilot testing and then several iterations in which the first version (1.0) was implemented with 200 expecting families. Quantitative indicators and qualitative feedback from participants and Navigators led to the design of Version 2.0, which was implemented with 212 expecting families. Again, quantitative indicators and feedback led to the design of Version 2.1, which was implemented with 194 expecting families. Versions 2.0 and 2.1 of CN are currently being implemented and tested via a randomized controlled trial (RCT) in a mid-sized community with a high rate of poverty. Here we report the implementation of CN during the prenatal period with families of all three versions as a way of describing the process of intervention design and iterative refinement before any impact findings become available.

## Methods

This prenatal implementation study is part of a longitudinal RCT of the CN program. For the RCT, pregnant individuals were recruited from Duke University prenatal care clinics, the Durham County Health Department, and a Fetal Diagnostic Center. Potential participants were identified using medical records and recruited by a clinical research coordinator when they attended their prenatal visits at the participating OB/GYN practices. Pregnant individuals were eligible for the study if they (1) received prenatal care at Duke University-affiliated prenatal care clinics, the Durham County Health Department, or the local Fetal Diagnostic Center during a regularly scheduled prenatal visit or ultrasound; (2) if their primary language was English or Spanish; and (3) were at or before 38 weeks gestation. Individuals residing outside of Durham County were ineligible to participate in the study, as were individuals who did not speak English or Spanish as their primary language or were planning to move out of the county within the next 2 months at the time of recruitment. Individuals were also ineligible if they had a cognitive impairment that impacted their ability to consent/participate. Those with current mental health conditions or active substance use disorders were not excluded from the study given the goal of the CN intervention to provide universal support to families. All intervention and RCT procedures were approved by the Duke University Health Institutional Review Board (IRB). This trial has been registered in ClinicalTrials.gov (NCT04438031).

Development and piloting of CN began in 2018, and the first version of the intervention (Navigation 1.0) was finalized in 2021. As reported below, CN has undergone several rounds of implementation and continuous quality improvement efforts. Here we report the implementation findings from each of the three versions tested to date: Navigation 1.0 (2021–2022), 2.0 (2022–ongoing), and 2.1 (2023–ongoing).

### Community Navigation Program

**Fig. 1:**
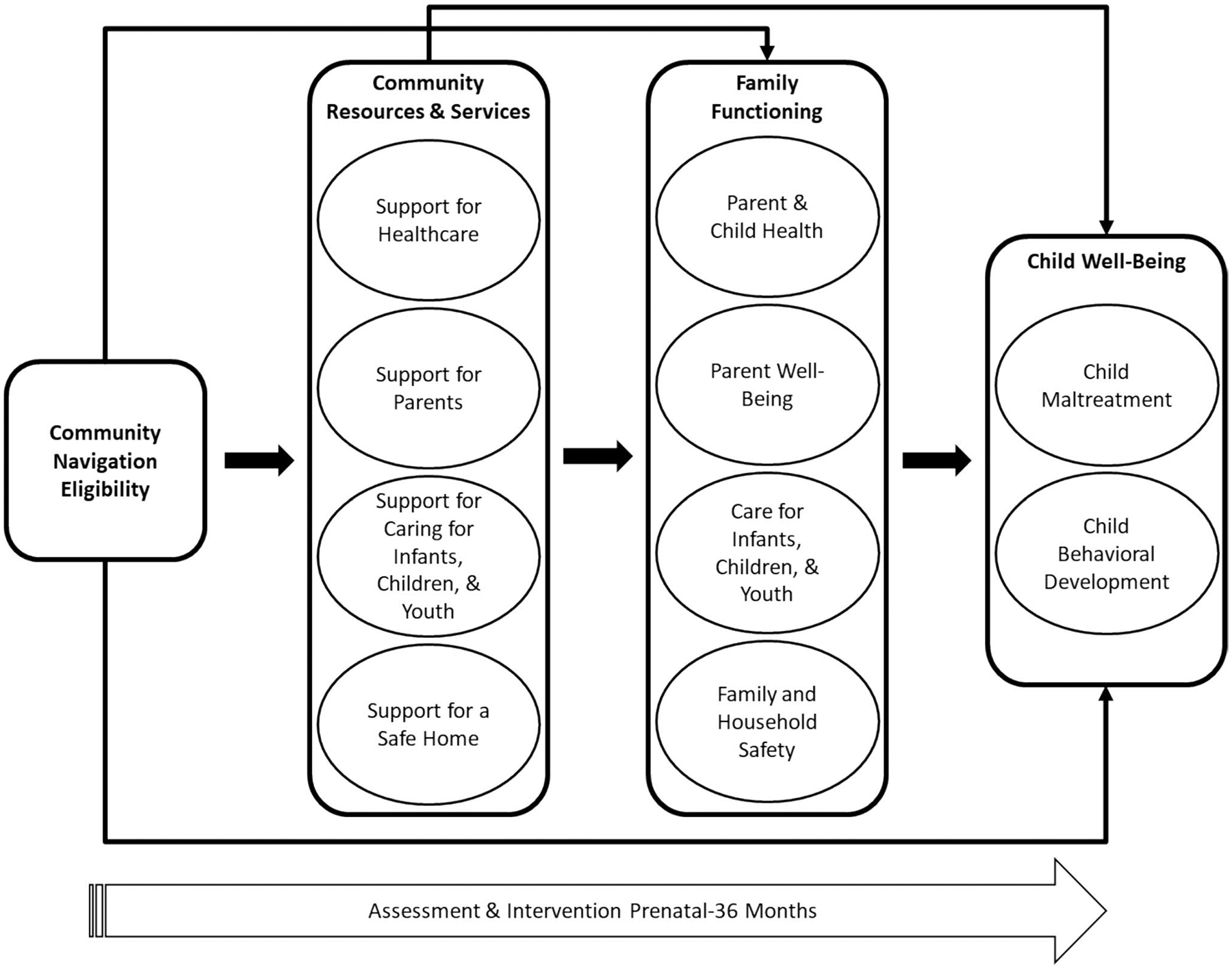
Community navigation logic model

Partnering with OB/GYN and pediatric clinics, CN uses the structure of prenatal care and pediatric well-child check-ups to reach families. After a pregnant individual consents to the study during a prenatal care check-up, a trained bachelor-level Navigator reaches out in person or by phone to offer congratulations and support beginning immediately and lasting through kindergarten matriculation, starting with the opportunity to receive 1–3 in-person or virtual visits to discuss supports the family might find helpful during pregnancy (Fig. 1). The participant is invited to include a partner, if a partner is present. During the initial prenatal visit, the Navigator assesses family strengths and needs in four domains, each containing three factors: (1) *Health Care* (caregiver health, infant health, health care plans); (2) *Caring for the Infant* (childcare plans, parent–child relationship, management of infant crying); (3) *A Safe Home* (household safety and material supports, family and community violence, history of parenting difficulties); and (4) *Parenting* (parent well-being, substance use, parent emotional support). Each factor is scored 1 if the family is functioning well with no need for intervention, 2 if a need is identified but can be addressed by the Navigator with education during the 1–3 visits, 3 if the need warrants a connection to a community service, and 4 if the risk is so imminent that intervention is necessary within 24 h. The Navigator also invites the family to nominate needs not yet discussed.

The Navigator and family then co-develop an intervention plan that might include one or more community connections to address family needs. Types of connections vary, such as mental health providers, substance abuse programs, housing loans, couples counseling, food stamps, and more. The Navigator helps the family connect with the agency and communicates with the agency provider to ensure that the connection is made. The Navigator follows up with the family one month later to review the experience. During the family’s third trimester, the Navigator re-contacts the family to reinforce connections to community services, identify any new family needs since the previous contact, and refer the family to Family Connects for postpartum support.^1^ This check-in acknowledges the rapid changes families may face during pregnancy and offers additional connections and resources as their needs may be different than earlier in the pregnancy. The Navigator also sets the stage for later Navigation touchpoints after the birth of the child.

### Navigator Expertise, Training, and Tools

The tools and protocols developed for CN require a deep set of skills, supported by ongoing training, to effectively engage a diverse population of families and the community partners who can support them. All Navigators were required to have at least a bachelor’s degree and two years of progressive experience in human services in the public sector. A Navigation Implementation Workgroup developed a set of recommended competencies to enable successful engagement of families and effective implementation of CN. These core competencies include cultural humility, building trust and engagement, being relationship-focused, having knowledge of maternal and child health and child development, having a knowledge of social drivers of health and health equity, understanding system coordination and community services, being partnership-focused, having direct service skills, maintaining ethical and professional behavior, and having experience with standardized assessments.

To support Navigators in their task of identifying community services to address family needs, a Community Alignment Specialist worked with community partners that serve perinatal families to develop an Agency Finder. The Agency Finder is an electronic directory of available community resources that is updated regularly. This directory describes each local agency’s goals, practices, eligibility, fees, waiting list, and evidence base. Regular review of the Agency Finder involves identifying gaps and changes in community services needed by families as well as expanding to include new formal and informal community services that address family needs.

### Intervention Quality Control

The intervention program was manualized and delivered consistently across families. Adherence to protocol as specified in the manual was assessed by having two independent experts complete quarterly fidelity and reliability checks during which they listened to audiotapes of CN visits for each Navigator. From a list of necessary program elements, the experts checked adherence (or not) to each model element. The experts also scored risk for each of the 12 factors to compare to the Navigator’s score.

The key categories of implementation measures in the process of designing CN include the following: (1) the *reach*, defined as the proportion of the eligible population that initiates participation through at least one home visit, the proportion of home-visited families that completes the intervention, and the net total participation; (2) *family engagement*, defined as the proportion of all participating families that co-identify with the Navigator a family need and the proportion of that group that gets connected to a community resource; (3) *family satisfaction*, captured from post-intervention interviews with families; and (4) *fidelity of implementation*, defined as the proportion of adherence to the manualized protocol. The last of these measures was collected only for the final Version 2.1.

## Results

### Version 1.0 Implementation

#### Recruitment

Families were approached during their prenatal OB/GYN visit to see whether they would like to participate in a research study of an intervention trial, until 400 families were consented. After 505 families were contacted, the goal of consenting 400 families to the study was reached, yielding a consent rate of 79%. Of the 400 families, 200 were randomly assigned to the Navigation 1.0 intervention, and 200 to control. The sample was diverse (43.2% Black, 32.7% White, 3.1% Asian American, 8.0% more than one race, and 13.0% unknown/unreported; 22.8% Hispanic or Latino/a) and representative of the Durham County birthing population. The intervention and control groups did not differ significantly across race, ethnicity, and age.

#### Visit Completion

Among the 200 intervention-assigned families, 160 (80%) successfully completed the initial prenatal visit to identify family needs. Of these 160 families, 137 (86%) completed all touchpoints and follow-up visits through birth, with a net full completion rate of 69%. Most non-completions were due to giving birth before all touchpoints could be completed. Of note, for families that consented to participate, Hispanic families were less likely to complete the prenatal touchpoint (65%) relative to non-Hispanic families (84%). However, no differences were seen across race [*χ*^*2*^(1) =.12, *ns*].

#### Identification of Needs and Community Connections

Of the 160 families for which an assessment of family needs was completed, 127 (79%) identified at least one family need (at least one score ≥ 2) that the Navigator tried to address. This rate is only slightly lower than the 94% rate found in previous FC trials with families at birth and indicated that families are willing to identify needs during pregnancy. However, only 7 families (4%) identified a need as “major, requiring connection with a community agency,” in contrast with the 45% in previous FC trials (Dodge et al., 2014, 2019), suggesting significant under identification of actual family needs for support and the necessity to revise the protocol in the next version.

#### Family Satisfaction with the Version 1.0 Navigation Program

At the final prenatal touch point, families provided feedback about the intervention. Among those who completed the touchpoint (n = 137), 97% said they felt supported by their navigator, 91% found the navigation to be helpful, and 98% said they would recommend it to other families. These high rates of satisfaction indicate the Navigators and the protocol were successfully connecting with families.

#### Navigation 1.0 Conclusions and Quality Improvement

Review of the protocol, interviews with Navigators, and consultation with community partners led to the conclusion that the protocol needed to be revised to seek more information about family needs, to allow families to report current challenges, and to connect more families with community resources.

### Version 2.0 Implementation

In response to the feedback from the Version 1.0 trial, the Navigation protocol was revised to solicit information about a wider range of family needs. Revisions included adding questions and promotional messages to aid Navigators’ support of families and assessment of their strengths and needs. A prenatal matrix guide was also created to provide in-depth guidance for scoring each domain and factor, as well as guidance for the appropriate Navigator responses for each (e.g., helping a mother schedule a medical appointment when a matrix score of 3 is given for an identified maternal health need). The matrix guide also includes several examples from Navigator experience to help guide scoring and responses. Overall, the scoring criteria (especially the distinction between a modest need scored as “2” and a major need scored as “3”) were revised to include a larger number of families as needing a community connection. Additionally, a formal referral process was created to connect families from CN to Family Connects for postpartum support.

Given the additions to the Navigation protocol, which would require more time devoted to each participating family, the Navigation team was expanded to include a Navigation supervisor and one additional full-time Navigator. Navigators also began participating in a weekly case conference led by the Navigation supervisor (a BA trained paraprofessional with over five years of experience in human services in the public sector). Additional expertise at case conference was provided by CN implementation leadership, including a developmental psychologist (Ph.D.), a pediatrician (M.D.), and a community alignment specialist.^2^ Case conference provided the opportunity for Navigators to discuss specific cases to hone their process in identifying needs, connecting to appropriate resources, and ensuring alignment in scoring family needs. The Navigation program manual was revised and labeled as Navigation 2.0. A new trial began with Navigation 2.0 (and continued with Navigation 2.1) in 2022 with a goal to recruit 800 new families and randomly assign 400 to intervention and 400 to control.

#### Recruitment

Once the revised Navigation trial began, families were approached during their prenatal OB/GYN visit to see whether they would like to participate in the study. Overall, 608 families were deemed eligible and approached for participation in the study of Navigation 2.0. Of those, 420 families consented,^3^ yielding a consent rate of 69%. Consented families were randomized to the Navigation 2.0 intervention (*n* = 212) or a control condition (*n* = 208). The 2.0 sample was 38.8% Black, 35.9% White, 5.7% Asian American, 3.3% more than one race, 1.0% other race, and 15.3% unknown/unreported; 20.1% Hispanic or Latino/a. The intervention and control groups did not differ significantly across race, ethnicity, and age.

#### Visit Completion

For prenatal Navigation 2.0, of the 212 families assigned to CN intervention, 126 (59%) successfully completed the initial navigation visit to identify family needs. Of these 126 families, 82 (65%) completed at least one follow-up visit prior to birth, yielding a net full completion rate of 39%. Of note, 93% of families who did not complete the prenatal follow-up or third trimester touchpoint gave birth prior to their scheduled visit, suggesting that initial recruitment timing was too late or that follow-up procedures were inadequate.

#### Visit completion varied by family demographics

Among families that consented to participate, Hispanic families were more likely to complete the prenatal touchpoint (78%) relative to non-Hispanic families (57%). However, across all races, White families were significantly more likely to participate than non-White families [*χ*^*2*^(1) = 13.60, *p* <.001]. This difference was primarily due to low completion rates of Black families. Only 42% of consented Black families completed the prenatal initial visit relative to 75% of consented White families.

#### Identification of Needs and Community Connections

Of the 126 families that completed an assessment of needs, 115 (91%) identified at least one family need (at least one score ≥ 2) that the Navigator tried to address during prenatal Navigation. This rate is higher than for Version 1.0 and is comparable to the 94% rate found for families at birth in previous FC trials (Dodge et al., 2014, 2019). Additionally, 76 (60%) of families identified at least one major need best supported by a referral to a community resource, a rate that was substantially higher than for Version 1.0 and higher than the 45% in previous FC trials. Nearly 300 referrals to community agencies were made based on identified family needs (average = 1.4 referrals per family), with a 45% successful connection rate within 1 month of the initial referral date. Of the 212 families assigned to CN, 73 families (34%) later completed a home visit with a Family Connects nurse after birth. Overall, these findings indicate that the Navigation Version 2.0 was successful in leading most families to identify needs that could be addressed through community connections.

#### Family Satisfaction with the Version 2.0 Navigation Program

Of the intervention-assigned families who completed the final prenatal touchpoint (n = 70), 100% said they felt supported by their navigator, 98% found the navigation to be helpful, and 98% said they would recommend it to other families.

#### Navigation 2.0 Conclusions and Quality Improvement

The iterative nature of the Navigation program development is consistent with the way that intervention program development has proceeded for other programs, including Family Connects. The poor metrics for community connections for Version 1.0 were disappointing but very helpful in revising the program. The comparatively high rates for the identification of family needs and community connections during the implementation of Version 2.0 were encouraging.

However, despite the high consent rate and increasing community connection rate, Navigation 2.0 had lower completion rates than Version 1.0 (e.g., only 59% completed the 2.0 initial prenatal visit relative to 80% for Version 1.0). The improved quality of family assessment and community connections appeared to be offset by poorer family completion rates, with a concerning low rate of completion among Black families. Low participation is particularly concerning in universal programs, as it reduces the potential for population-wide impact.

### Version 2.1 Implementation

Further refinements to the prenatal Navigation protocol were made to address the low completion rates, particularly among Black families, while still maintaining high quality of family assessment and community connections. These refinements were introduced and tested in Navigation 2.1:

1. Enrollment criteria: The first implemented change was to alter the gestational age enrollment criteria for the RCT. In Versions 1.0 and 2.0, pregnant families were eligible for recruitment and consent up to 38 weeks gestation. As we learned in Version 2.0, recruitment so late in the pregnancy did not provide enough time for Navigators to initiate services and follow-up with families before delivery, particularly given the longer, more intensive nature of the 2.0 protocol relative to the 1.0 protocol. As noted previously, only 39% of families completed either the 4-week follow-up or third trimester touchpoints. Of the families that did not complete either touchpoint, 93% delivered prior to completion. Therefore, to ensure that the Navigators had sufficient time to provide services and support families during the prenatal period, the enrollment criteria were changed, such that pregnant families were eligible for 2.1 until they reached 29 weeks gestation. Although the future CN task of reaching families who do not enter prenatal care until after 29 weeks remains, we restricted enrollment in prenatal CN to 29 weeks gestation, both to encourage early enrollment and to preserve the full integrity of this intervention.
2. Contact procedures: To further ensure that all consented families had an opportunity to receive Navigation services, changes were made in 2.1 for contact procedures. Specifically, Navigators were instructed to contact all newly enrolled families within 24–48 h of consent using multiple forms of contact (email, text, and phone call). Additionally, all three forms of contact were to be used weekly until all families had been successfully contacted.
3. Participant tracking: Finally, examination into our contact attempts (all attempts were logged by Navigators for continuous quality improvement efforts) indicated that in Version 2.0, some families experienced long gaps between contacts. For example, after completing the initial prenatal visit, some families were not contacted again by the Navigator until months later (thereby missing their window for the 4-week follow-up and, for some, missing their opportunity to participate in any further prenatal support due to delivering their child). These gaps in contacts were judged to be due in part to inefficient participant tracking mechanisms. As such, our team revised the participant tracking logs to include status indicators (e.g., scheduling in progress, initial visit scheduled, 4-week follow-up scheduled, all visits completed). These status indicators were color-coded and automatically tabulated to ensure that the Navigation Supervisor could check statuses in real time and monitor any lags in scheduling or visit completion. Challenges with participant engagement were discussed and addressed weekly during Navigator supervision meetings to ensure that all efforts were made to engage families prior to delivery.

In addition to the changes made to improve completion rates, we also revised Navigation 2.1 to standardize Navigator training and skills. Our team created a standardized training program for all Navigators, who then underwent two months of additional training prior to implementing Navigation 2.1 (see Supplementary Materials). After these changes were implemented, the trial continued with the recruitment of Navigation 2.1 families in 2023.

#### Recruitment

For Navigation 2.1, 715 families were deemed eligible and approached to participate in the research RCT, and 395 (55%) consented. Of the 395 enrolled families, 194 were randomly assigned to intervention and 201 as controls. The sample was diverse (34.2% Black, 45.1% White, 7.3% Asian American, 1.0% more than one race, and 12.4% unknown/unreported; 27.5% Hispanic or Latino/a). Average gestational age at time of consent was 23 weeks 9 days (gestational age at time of consent was not tracked for Versions 1.0 or 2.0).

#### Fidelity

Systematic recording of fidelity was introduced for this version. Two independent observers with Ph.D./M.D. training and 10 + years of program implementation and evaluation experience listened to video and audio recordings for all 6 Navigators to check for adherence to manualized action steps and agreement on scoring of each of 12 risk/need factors. Fidelity checks were completed quarterly for each Navigator. Observer-rated adherence to the manual was high: 92.9% (386 of 415 program elements checked). Interrater agreement on scoring of risk yielded a mean κ coefficient across the 12 risk factors of 0.67 (coefficients > 0.60 are considered substantial; Landis & Koch, 1977).

#### Visit Completion

Of the 194 intervention-assigned families, 153 (81% of eligible participants) successfully completed the initial prenatal Navigation visit to identify family needs. Of these 153 families, 126 (82%) completed at least one follow-up visit prior to birth, yielding a net full completion rate of 65% (Table 1). Reasons for not completing the initial prenatal Navigation visit included declining the visit (*n* = 2; 1%), becoming ineligible or being withdrawn from the study (e.g., moving out of county or experiencing pregnancy loss; *n* = 4; 2%), or giving birth prior to completing a visit (*n* = 35; 18%). In contrast to Versions 1.0 and 2.0, visit completion rates did not differ by race or ethnicity [ethnicity: *χ*^*2*^(1) =.50, *ns;* race: *χ*^*2*^(1) = 1.81, *ns*]. In particular, Navigation 2.1 saw a large increase in participation of Black families (70%) relative to Navigation 2.0 (42%). Overall, visit completion rates for Navigation 2.1 suggest that Navigators were largely successful in engaging families across all racial and ethnic identities.

#### Identification of Needs and Community Connections

Of the 153 families that completed an assessment of needs, 150 (98%) identified at least one family need that the Navigator addressed during prenatal Navigation (at least one score of 2 or higher). Specifically, no families (0%) scored 4 at least once, indicating the need for immediate emergency intervention; 114 (75%) scored 3 (but not 4) at least once, indicating a serious need best served by referral to a community agency; 36 (23%) received a 2 (but not a 3 or 4) at least once, indicating a need that was addressed by brief Navigator intervention or the provision of educational materials; and 3 (2%) received the lowest need score (1) in all 12 factors.

The highest factors of need were *Health Care Plans* (30% had a score of 3 indicating a major need, generally due to not having a primary care physician or pediatrician), *Household Safety and Material Supports* (28% had a major need, primarily due to food insecurity or not having a crib/car seat for their baby), and *Childcare Plans* (26% had a major need of not having a childcare plan in place once baby arrives). Strikingly, 20% of pregnant participants who completed the initial prenatal Navigation visit screened positive for mental health concerns related to depression and/or anxiety and were referred to a community resource for mental health support. Another 30% of participants were already in treatment for mental health concerns and/or reported experiencing depression/anxiety in the past.

Among the 114 families with at least one identified major family need requiring a community connection, 279 referrals to community agencies were made (average = 1.8 referrals per family), with an additional 62 referrals being made for resources unrelated to needs but based on family request. For some referrals, families wished to self-refer (*n* = 33 referrals), declined the referral (*n* = 9), already had a resource/service in place to meet the need (*n* = 20), or the ideal resource was not found (*n* = 3). Additionally, Navigators provided educational materials or resources for 52% of families. The most common educational materials pertained to mental health (e.g., signs of postpartum depression), emotional support (e.g., lists of local parenting groups), and parent–child relationships (e.g., list of local breastfeeding or parenting classes).

During 1-month follow-up contacts, families reported on their community connections. Of the 114 families who received a community referral, 75 (66%) reported being successfully connected to at least one referral organization. This figure means that 49% of all families (75 of 153) that participated in CN ultimately became connected to a community agency matched to their identified family needs. A successful connection had been established with a community service provider for 51% (126/246) of all referrals. The most commonly cited reasons for not connecting with a referral resource included lack of time, the service no longer being needed, and families prioritizing other needs first. Finally, of the 194 families assigned to CN, 91 families (47%) completed a home visit with a Family Connects nurse, a marked improvement over Navigation 2.0.

#### Family Satisfaction with the Version 2.1 Navigation Program

Of the 120 intervention-assigned families who completed the final prenatal touchpoint, 100% said they felt supported by their Navigator, 98% found Community Navigation to be helpful, and 98% said they would recommend it to other families. Example feedback from families who participated in Navigation 2.1 included, “I have a sense of relief that someone is checking on me. Someone is asking how I am doing in all areas of my life. Emotionally, especially.” One mother stated, “The calls with you are comforting. They make me feel that someone is paying attention to how I feel and what I am thinking or wondering. It is nice to feel that someone is worried about you.” Finally, as another parent put it, “I enjoy having someone to talk to who is not judgmental or going to give their own opinion. That’s really great about the program. Being a first-time parent can be really difficult because there is so much information out there, but your program takes the legwork out of making connection to the things I need.” These reports of high satisfaction indicate that the Navigators and the protocol were successful in connecting with and supporting families.

#### Navigation 2.1 Conclusions and Quality Improvement

Overall, implementation metrics from Navigation 2.1 demonstrate marked improvement over Versions 1.0 and 2.0 (Table 2). Regarding visit completion rates, 81% of Navigation 2.1 families completed the initial prenatal visit relative to 59% of Navigation 2.0 families. Additionally, 65% of Navigation 2.1 families completed at least one follow-up touchpoint, an improvement from the 39% of Navigation 2.0 families. Importantly, no differences in completion were found across race and ethnicity, indicating that Navigation 2.1 was the first version of CN to successfully engage families at high rates across all groups.

With respect to identification of needs and connections to community resources, the percentage of families reporting at least one need increased from 91% in Navigation 2.0 to 98% in Navigation 2.1, the percentage of families that had a major need best met by a community resource increased from 60 to 75%, the average number of referrals per family increased from 1.4 to 1.8, and the connection rate increased from 45 to 51%. Overall, these trends suggest that the quality improvement efforts across Navigation versions led to an improved protocol that more effectively served families and supported their needs during pregnancy.

## Discussion

Community Navigation is a novel approach to supporting birthing families’ psychosocial needs during the prenatal period. This approach includes population reach, universal preventive interventions, screening for needs, and matching of community resources to meet family-specific needs. As reported, the Navigation protocol underwent multiple rounds of quality improvement, implementation, and testing. Quantitative data and consumer feedback were used iteratively to reach successful implementation. Findings from the final version, Navigation 2.1, indicate that this program can be implemented during the prenatal period with high levels of engagement, identification of needs, connections to community services, and family satisfaction.

Crucial gaps exist in the systems of care for families of young children. Beyond pediatric medical system supports for health-related needs, parents in the US are largely left on their own to find support and meet their child’s needs until kindergarten matriculation—at which point, the formal public education system provides some vigilance and supports access to local resources and services as needed. Our findings from prenatal CN implementation demonstrate high rates of needs for nearly all families. After revising the Navigation protocol to better identify family needs, we found that nearly all families (98% in Version 2.1) reported at least one need during the prenatal period. Additionally, most families had a major need that was best met through a referral to a community resource (75% in Version 2.1); the remaining 23% of families had needs that the Navigator could address through brief intervention or education. These rates are comparable to those reported in the Family Connects trials with families just after birth (Dodge et al., 2014, 2019) and stress the importance of universal support to families across the birthing period. Bringing a child into the world is a transformative experience and, as such, virtually all families can benefit from support during this time. However, no integrative, continuous system exists to provide community-level support for early child behavior and development, or to support family needs more broadly. Early childhood home visiting steps into this gap to provide support to a small number of families, typically those eligible due to demographic risk.

Across all Navigation 2.1 families, the areas of highest need were health care plans, household safety and material support, and childcare plans. Nearly a third of families had a need related to health care plans, generally due to not having a primary care physician for the mother or a pediatrician selected for the child. This lack of connection to health care providers was typically due to lack of knowledge about how to get Medicaid or other health insurance, lack of transportation or easy access, or discomfort with assigned providers. The current health care delivery system is passive in addressing these barriers and often relies on families coming forward on their own. Both adults and children often face major barriers to receiving routine primary medical care (Trinoskey-Rice et al., 2023; Wells et al., 2024). Nearly a third of all adults and 1 out of 10 children lack a primary care physician, and these numbers are increasing. Low-income families receiving Medicaid are particularly hard pressed, as the number of pediatricians is declining due to growing financial obstacles, resulting in long waitlists or an inability to locate a pediatrician willing to accept new patients (Wells et al., 2024). The Navigators in the present study worked with families to identify primary care physicians and pediatricians who accepted their insurance or provided care to those without insurance, so that families could receive routine preventive care. When barriers to service receipt were encountered (e.g., waitlists, language limitations), the Navigators utilized the Agency Finder and the expertise of the team’s community alignment specialist to identify alternative resources or alternative methods for connecting the family to needed support.

Similarly, many families found themselves struggling to provide the basics needed to live and work in the current economy. Nearly a third of 2.1 Navigation families had a major material need, including food insecurity or not having access to essential infant care materials, such as cribs or age-appropriate car seats. Material hardship is a growing concern among families with young children. A recent survey found that half of children in the US live in households experiencing financial difficulties (United For ALICE, 2024). Importantly, although only 16% of children live below the federal poverty line, an additional 34% live in households that do not make enough money to afford the basics in their communities (i.e., housing, childcare, food, transportation, health care, technology, and taxes). Navigators were able to connect families to available resources in the community to obtain free or discounted car seats and cribs, enroll in government assistance programs (e.g., food stamps, housing vouchers), and access food pantries.

Childcare availability and costs are also of major concern to many families. Today, almost all parents of young children need to, or want to, work outside the home (Herbst, 2023). Many families of infants have difficulty finding suitable care, often due to long waitlists which require application well before birth. We found that nearly a third of 2.1 Navigation-assigned families struggled during pregnancy to locate affordable, high-quality childcare for their newborn. Childcare costs have increased dramatically over the past 40 years and are now one of the leading expenses faced by parents (Herbst, 2023; Tekin, 2021). Childcare costs are particularly prohibitive for low-income families—low-income parents spend nearly 30% of their income on center-based care, compared to 8% of income for middle-to-high-income parents (Tekin, 2021). Moreover, the quality of childcare varies widely across providers and sectors, furthering inequality and disparities in child outcomes. In CN, families were supported by their Navigators to apply for childcare vouchers (many families did not know how to apply or whether they were eligible for support), locate options near their homes or work, and/or apply for the state-operated Pre-Kindergarten program for older siblings to reduce childcare costs.

It is also worth noting the incredibly high rates of perinatal depression and anxiety reported by mothers during the Navigation visits, in alignment with other reports (Murthy, 2024). Twenty percent of mothers screened positive for perinatal depression or anxiety, while an additional 30 percent were already in treatment and/or had experienced mental health challenges in the past. The high need for mental health support was consistent across socioeconomic status and demographic characteristics, providing further evidence for universal family support. Previous work demonstrates that perinatal anxiety and depression tend to increase over the course of pregnancy (Cheng et al., 2021). Moreover, about half of all pregnant and new mothers report symptoms of anxiety or depression. These trends highlight the crucial need for continued support across pregnancy and the postpartum period, especially given the numerous impacts that maternal mental health can have on the mother, the child, and the family (Junge et al., 2017; McGovern et al., 2022; Sweeney & Wilson, 2023). One goal of the CN program is to ensure that all parents have access to mental health support as needed. Over time, as Navigation-assigned families complete later touchpoints (e.g., at child ages 12 months, 24 months, 36 months), we will examine whether mental health needs decrease as more families are connected to resources.

Despite the high rates of engagement, identification of needs, and connections to community resources, our findings also suggest areas for ongoing quality improvement efforts. For example, while Navigators were able to help families connect to half of all referrals (Version 2.1), barriers to service connection remain and should be addressed to the extent possible. The most commonly reported barriers were lack of time, the service no longer being needed, and families prioritizing other needs first. These reasons suggest that additional training may improve the process by which Navigators refer families to community resources. First, the referral plan is always co-developed with families. For families with multiple needs, Navigators must be trained in how to collaborate with families in prioritizing referrals to case management or other services capable of working with families over an extended period of time on multiple areas of need. Once connections have been made to ongoing support programs, Navigators can coordinate with those support providers to ensure that all family needs are being addressed. Secondly, Navigators should be trained to use motivational interviewing techniques (Rollnick & Miller, 1995) to explore readiness for change in areas known to be associated with excess risk (e.g., when a mother has an unaddressed mental health need). These process improvements are being implemented and tested at the later Navigation touchpoints.

We reach three general conclusions. First, the demonstrated high rate of diverse unmet needs experienced by birthing families suggests a huge public policy demand to increase the array of services and resources that should be made available to families based on specific needs. Second, a deliberate process of quantitative data collection coupled with qualitative feedback from families and Navigators can lead to substantial improvement in the design and delivery of a universal family support program. We recommend a similar process for other endeavors. Third, the brief, universal prenatal CN program can be delivered with high penetration, fidelity, engagement, connection to community resources, and family satisfaction. Data are currently being collected via the Navigation RCT to test the impact of the program on family and child outcomes. Future reports will include impact findings, as well as implementation metrics from additional Navigation time-points (12, 24, 36, 48, and 60 months). Overall, Community Navigation holds promise as a system of universal primary psychosocial care for birthing families.

## Supplementary Material

Supplement

The online version contains supplementary material available at https://doi.org/10.1007/s11121-026-01939-7.

## Figures and Tables

**Table 1:** Prenatal navigation implementation metrics for Version 2.1 (*N* = 194)

	Initial prenatal visit	4-Week follow-up	3rd Trimester follow-up
Completed	153 (79%)	115 (59%)	120 (62%)
Not completed			
Declined	2		
Became ineligible/withdrawn	4		
Gave birth prior to visit	35		
Referrals			
Total referrals	269		
Average number of referrals per family	1.8		
Referral connection rate	51%		
Families connected to at least 1 resource	66%		
Matrix scores			
% Families w/a highest score of 4	0%		
% Families w/a highest score of 3	75%		
% Families w/a highest score of 2	23%		
% Families w/lowest need score (1) across all factors	2%		

**Table 2:** Prenatal navigation implementation metrics by version

	Navigation 1.0	Navigation 2.0	Navigation 2.1
Consented	400	420	395
Control	200	208	201
Navigation	200	212	194
Completion rates for navigation-assigned families			
Initial prenatal visit	80%	59%	79%
Follow-up	69%	39%	65%
Need scores			
% Families w/at least one major need scored as 3	4%	60%	75%
% Families w/at least one need scored as 2 or 3	79%	91%	98%
Family satisfaction with community navigation			
Family satisfaction (found program helpful)	91%	98%	98%

## Data Availability

Data are not publicly available due to the ongoing CN trial. Individual data requests should be submitted to the first author.

## Footnotes

1 Family Connects is a separate program from CN. Currently, in Durham County, Family Connects is provided by an independent non-profit organization. During Navigation 1.0, the two programs operated independently and families participating in CN were not directly referred to Family Connects. During Navigation 2.0 and 2.1, an effort was made to more purposefully partner the two programs. As such, Navigators referred any prenatal family who gave their consent to Family Connects.

2 The community alignment specialist (CAS) ensures that the Navigators have accurate contact and capacity information for relevant community agencies and works actively to identify gaps in supports needed by families. Effective coordination by the CAS requires strong relationships with medical providers, community agencies, and other stakeholders in the community.

3 For Navigation 2.0 only, some families (*n* = 34) were recruited via phone after their prenatal visit rather than in person during the visit due to temporary staffing limitations of the recruitment team.
