# Supplementary material for: Community Navigation: Iterative Development and Implementation of a Prenatal Psychosocial System of Care
Source: Prev Sci. Author manuscript; Available in PMC 2026 Jul 14. (PMC13360660; doi:10.1007/s11121-026-01939-7)
Supplement: Supplement [file NIHMS2193535-supplement-Supplement.docx]

**Supplement: Navigation Core Competencies and Training Guide**

**Navigation Core Competencies**

The tools and protocols developed for Navigation require a deep set of skills, supported by ongoing training, to effectively engage a diverse population of families and the provider partners who can support them. A Navigation Implementation Workgroup developed a set of recommended competencies that will enable successful engagement of families and effective implementation of Navigation.

| Core Competency | Training and/or Educational Material |
| --- | --- |
| Cultural Humility  The ability to maintain a humble and respectful attitude toward individuals of diverse cultures. | **Lesson 9: Cultural Considerations (35 mins)** <https://learn.nctsn.org/>  *Create an account and search for Attachment Vitamins Course. Then select Lesson 9 of Attachment Vitamins.  **Online Implicit Bias Test**  <https://implicit.harvard.edu/implicit/takeatest.html> |
| Building Trust and Engagement  The ability to quickly build trust and rapport with diverse families and engage them in productive dialogue. Skills that contribute to this core competency include trauma-informed care and motivational interviewing. | **Trauma-Informed Care**  **Trauma Toolkit**  <https://ronhuxley.thinkific.com/courses/traumatoolbox>  **Motivational Interviewing**  Motivational Interviewing Webinar (3 Part Series)  Idaho Department of Health & Welfare  Part 1 (62 mins): <https://www.youtube.com/watch?v=Cu_Ojsy-cQk>  Part 2 (57 mins): <https://www.youtube.com/watch?v=Dz4v_-4qqjQ>  Part 3 (54 mins): <https://www.youtube.com/watch?v=kptUqYlYeWs> |
| Relationship-Focused  The ability to establish relationships with families in a short amount of time, following their lead and using a collaborative approach | This core competency will be largely supported via on-the-job training, including reflective supervision and weekly case conferences with the navigation team. |
| Maternal and Child Health & Child Development  The ability to apply a general working knowledge of health and development topics | **What Is Early Childhood Development? A Guide to the Science**  Center on the Developing Child at Harvard University  <https://developingchild.harvard.edu/guide/what-is-early-childhood-development-a-guide-to-the-science/> |
| Social Drivers of Health & Health Equity  The ability to recognize how Social Drivers of Health can impact an individual’s and a community’s well-being and to understand the implications for inequity | **Social Determinants of Health**  Claire Pomeroy’s TEDxTalk  <https://www.youtube.com/watch?v=qykD-2AXKIU&vl=en>  **Adverse Childhood Experiences**  Nadine Burke Harris: “How childhood trauma affects health across a lifetime”  <https://www.ted.com/talks/nadine_burke_harris_how_childhood_trauma_affects_health_across_a_lifetime?language=en> |
| Understanding System Coordination & Community Services  The ability to support the development of an early childhood system of care | Community alignment training conducted by navigation team at hire. Also, continued professional development through weekly case conferences and supervision. |
| Partnership-Focused  The ability to work collaboratively with medical offices and external partners to establish the Navigator as a valued member of the care team | This core competency will be largely supported via on-the-job training, including reflective supervision and weekly case conferences with the navigation team. |
| Direct Service Skills  The ability to engage individuals and move them into action by providing clear and accurate resource information | Many of the skills required for this core competency are covered in other trainings (e.g., child health, cultural considerations).  Additional trainings on topics such as conflict management and problem-solving will be offered as needed by individual navigators. |
| Ethical and Professional Behavior  The ability to adhere to relevant ethical standards | **HIPAA Privacy and Security**  Duke LMS Annual Training (00156846- 15 mins)  <https://lms.duhs.duke.edu/>  *This training is required by Duke annually |
| Assessment  The ability to identify and address the strengths, needs, and risks of families | This core competency will be largely supported via on-the-job training, including reflective supervision and weekly case conferences with the navigation team. |
